# Conservation Patterns of HIV-1 RT Connection and RNase H Domains: Identification of New Mutations in NRTI-Treated Patients

**DOI:** 10.1371/journal.pone.0001781

**Published:** 2008-03-12

**Authors:** André F. A. Santos, Renan B. Lengruber, Esmeralda A. Soares, Abhay Jere, Eduardo Sprinz, Ana M. B. Martinez, Jussara Silveira, Fernando S. Sion, Vinay K. Pathak, Marcelo A. Soares

**Affiliations:** 1 Laboratório de Virologia Humana, Departamento de Genética, Universidade Federal do Rio de Janeiro, Rio de Janeiro, Brazil; 2 HIV Drug Resistance Program, National Cancer Insitute-Frederick, Frederick, Maryland, United States of America; 3 Hospital de Clínicas de Porto Alegre, Porto Alegre, Brazil; 4 Fundação Universidade do Rio Grande, Rio Grande, Brazil; 5 Hospital Universitário Gaffrée e Guinle, Rio de Janeiro, Brazil; 6 Unidade de Genética, Instituto Nacional de Câncer, Rio de Janeiro, Brazil; Instituto de Pesquisa Clinica Evandro Chagas, FIOCRUZ, Brazil

## Abstract

**Background:**

Although extensive HIV drug resistance information is available for the first 400 amino acids of its reverse transcriptase, the impact of antiretroviral treatment in C-terminal domains of Pol (thumb, connection and RNase H) is poorly understood.

**Methods and Findings:**

We wanted to characterize conserved regions in RT C-terminal domains among HIV-1 group M subtypes and CRF. Additionally, we wished to identify NRTI-related mutations in HIV-1 RT C-terminal domains. We sequenced 118 RNase H domains from clinical viral isolates in Brazil, and analyzed 510 thumb and connection domain and 450 RNase H domain sequences collected from public HIV sequence databases, together with their treatment status and histories. Drug-naïve and NRTI-treated datasets were compared for intra- and inter-group conservation, and differences were determined using Fisher's exact tests. One third of RT C-terminal residues were found to be conserved among group M variants. Three mutations were found exclusively in NRTI-treated isolates. Nine mutations in the connection and 6 mutations in the RNase H were associated with NRTI treatment in subtype B. Some of them lay in or close to amino acid residues which contact nucleic acid or near the RNase H active site. Several of the residues pointed out herein have been recently associated to NRTI exposure or increase drug resistance to NRTI.

**Conclusions:**

This is the first comprehensive genotypic analysis of a large sequence dataset that describes NRTI-related mutations in HIV-1 RT C-terminal domains *in vivo*. The findings into the conservation of RT C-terminal domains may pave the way to more rational drug design initiatives targeting those regions.

## Introduction

The HIV-1 RT is the enzyme responsible for synthesizing a double-stranded integrative cDNA from the single-stranded viral genomic RNA early in the virus life cycle. HIV-1 RT is a heterodimer composed of two subunits, p66 and p51; p51 is derived from the proteolytic processing and removal of C-terminus (codons 441 to 560) from p66 [1]. The p66 subunit comprises two major portions, the polymerase (codons 1 to 440) and the RNase H domains. The polymerase domain encodes the RT fingers, palm, thumb and connection motifs [2]–[4]. The RNase H domain promotes RNA degradation from the DNA:RNA duplex during reverse transcription. Other RNase H functions include the removal of the tRNA_3_^Lys^ and the removal of the polypurine tract (PPT), primers for the DNA minus-strand and DNA plus-strand synthesis, respectively [5]–[7].

The polymerase RT portion is currently targeted by two classes of ARV drugs, the nucleoside reverse transcriptase inhibitors (NRTI) and the non-nucleoside RTI (NNRTI). HIV drug resistance mutations in this domain are extensively characterized for these classes of ARV drugs [8]. To date, however, the influence of connection and RNase H RT domains in NRTI or NNRTI resistance is poorly understood. Currently the region of RT selected for sequencing in resistance studies ranges mostly from codons 1 to 250 [9], [10], and does not include these domains. Recently, Nikolenko *et al.* pointed out mutations in the RNase H domain that reduce its activity and concomitantly increase the resistance to thymidine analogues *in vitro* [11]. They have also proposed a new drug resistance mechanism where the lowered RNase H activity would allow more time for NRTI excision. This mechanism is also influenced by the connection domain, as point mutations in the region that contact template-primer affect RNase H activity [12]. In the present study we analyzed the connection and RNase H domains from treatment-naïve and NRTI-experienced patients infected with different HIV-1 subtypes in the search for structural conservation patterns and for putative new NRTI-related mutations. This analysis will also enable the establishment of conserved regions in the connection and RNase H RT domains, which could be an essential tool for future rational drug design initiatives targeting those domains.

## Methods

### Sample collection

Plasma samples from HIV-positive patients, both treatment-naïve and HAART-experienced, were collected from three Brazilian HIV/AIDS reference centers: two in Rio Grande do Sul (RS) state, Hospital de Clínicas de Porto Alegre and University Hospital of Rio Grande, and one located in Rio de Janeiro (RJ), Hospital Gaffrée e Guinle. Patients were recruited from 2002 to 2003 in RS and from 2004 to 2006 in RJ. All patients signed a written consent form for participating in the study, and the Internal Review Boards from all three Institutions have approved the study.

### RNA isolation, nested PCR and RNase H domain sequencing

HIV viral RNA was isolated from 50 µl of patient's plasma using Trizol, followed by cDNA synthesis with Moloney murine leukemia virus RT and random primers as previously described [13]. Viral RNase H domain was amplified by nested PCR using Platinum Taq polymerase (Invitrogen, CA, EUA). The first round was conducted using primers 5′tatacgtaagccacctggattc3′ (3762-3783 nucleotide positions relative to HXB2) and 5′cagtctacttgtccatgcatggcttc3′ (nt relative positions 4371-4396) with an annealing temperature of 55°C. The second round was done using primers 5′ggtaccagttagagaaagaaccca3′ (nucleotide positions 3826-3849) and 5′cattgcctctccaattactgtgatatttctcatg3′ (nt positions 4263-4295) with an annealing temperature of 56°C. A 469-bp fragment corresponding to RNase H codons was amplified, purified and sequenced in an automated ABI Prism 3100 Genetic Analyzer (Applied Biosystems, Foster City, CA, USA). Sequences obtained were assembled and manually edited using SeqMan (DNAStar, Madison, WI, USA).

### HIV-1 subtype characterization

To eliminate sample contaminations or mix-ups and to determine the isolates' subtype, a FASTA file with all edited sequences and representative sequences of all HIV-1 subtypes obtained from Los Alamos HIV database (http://hiv-web.lanl.gov) [14] was constructed. The HIV-1 subtype of each sample was determined through phylogenetic inference using the neighbor-joining method and Kimura 2-parameter model of nucleotide substitution in the MEGA 2.1 package [15]. One thousand replicates of bootstrap were performed to infer cluster robustness and the sequence of HIV-1 group O 99SE-MP1299 (GenBank acc. no. AJ302646) was used to outgroup the trees [16].

### Global thumb, connection, and RNase H domain sequences

For the conservation analysis of thumb, connection and RNase H domains and potential drug resistance mutations, we searched all available HIV-1 group M sequences spanning all subtypes (A–D, F–H, J and K) and CRF01_AE and CRF02_AG from the Los Alamos HIV database (http://hiv-web.lanl.gov) [14] and the Stanford HIV Drug Resistance Database (http://hivdb.stanford.edu/) [17]. Only isolates from patients for which treatment information was available, and which included the use of NRTI, were included in the analysis. The Stanford database already includes full treatment history for all sequences. For the Los Alamos database, ARV-experienced sequences had their GenBank accession number retrieved, which was used to track treatment history in the Stanford database. To prevent the use of more than one sample from the same patient, all sequences obtained were subjected to phylogenetic inference using the methodology described above, and for sequences shown to be phylogenetically close-related only one of them was kept.

### Determination of drug-related mutations

In view of potential sequence biases created from comparing different HIV-1 polymorphisms present in different subtypes, only subtype B sequences were used for drug-related mutation comparisons. Sequences belonging to subtype B from treatment-naïve and experienced groups were separately aligned using ClustalX [18] in the freely available program BioEdit v. 7.0.5.3 [19]. A subtype B consensus sequence from treatment-naive subjects was used as reference in the analysis. Nucleotide sequences were translated into amino acids and residue differences were compiled between treatment-naïve and NRTI-treated patients. All amino acid differences between both treatment groups were evaluated by statistical inference using two-tailed Fisher's exact test, and a *p*-value<0.05 was considered significant.

In an attempt to investigate the potential role of specific ARV drugs in selecting resistance mutations, we have analyzed 28 subtype B sequences from subjects that underwent monotherapy with AZT (the only drug for which there were cases of single drug exposure prior to sample collection). Those sequences were compared to the subtype B drug-naïve isolates described above for significance of amino acid residue frequencies.

### Structural visualization of HIV-1 RT domains

To facilitate the visualization of conserved positions and the inferred drug-related mutations, the atomic coordinates of the crystal structure of HIV-1 BH10 (subtype B) RT in contact with the DNA:RNA hybrid (PDB entry 1HYS) were used [20]. The software ViewerLite 5.0 was used to visualize the file and to color the conservation patterns and mutations which differ significantly between treatment-naïve and NRTI-treated groups.

## Results

### Conservation of RT C-terminal domains

Five hundred and ten sequences of thumb and connection domains (RT codons 298-440) were retrieved from the databases, of which 280 were treatment-naïve (HIV-1 subtype composition: 38 A, 72 B, 73 C, 34 D, 6 F, 4 G, 1 H, 1 K, 44 CRF01_AE and 7 CRF02_AG) and 230 were NRTI-treated (10 A, 188 B, 4 C, 4 D, 20 CRF01_AE and 4 CRF02_AG). For the RNase H domain (RT codons 441-560), we obtained 450 sequences, of which 276 were treatment-naïve (39 A, 66 B, 69 C, 35 D, 7 F, 5 G, 1 H, 1 J, 44 CRF01_AE and 9 CRF02_AG) and 174 were NRTI-treated (1 A, 153 B, 2 F and 18 CRF01_AE).

We successfully amplified and sequenced the RNase H domain (codons 441-560) of 118 isolates from Brazilian HIV-positive patients. The phylogenetic inference showed that half of them belonged to subtype C (n = 59) and the other half was assigned to subtype B (n = 59) (data not shown). Regarding ARV treatment status, 58 patients were naive (B = 27 and C = 31) and 60 were on treatment (B = 32 and C = 28). Together with the sequences from public databases, we gathered 334 RNase H sequences of drug-naïve and 234 RNase H sequences of NRTI-treated patients. For the RT connection domain, we have obtained 510 isolates from the public databases for further analysis.

**Figure 1:**
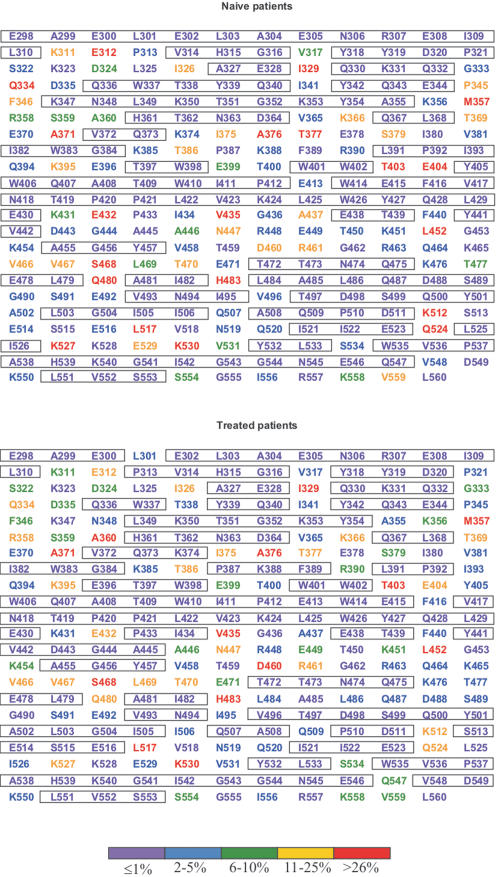
Analysis of conservation of HIV-1 RT connection and RNase H domains from HIV-1-infected drug-naïve and NRTI-treated subjects. Amino acid residues are represented as the group M consensus. Degrees of conservation are color-coded as shown at the bottom. Conserved motifs (variability below 1%) are boxed.

Sequences of RT C-terminal domains were aligned, subtyped by phylogenetic analysis (data not shown), and evaluated by their extent of codon polymorphisms (Figure 1). In drug-naive isolates, the number of non-variable sites (defined as sites exhibiting variation ≤1%) was 165 (63%), a similar number found among NRTI-treated isolates (163; 62%). In some cases, sites seemed more variable in the naïve isolates than in the NRTI-treated isolates. This is explained by the different subtype proportions among those two groups. While 26% of thumb and connection domain sequences from drug-naïve subjects were represented by subtype B, this proportion among treated subjects was of 82%. Similarly, subtype B accounted for only 28% of RNase H domain sequences from naïve subjects, while it accounted for 80% among treated subjects.

### NRTI-selected amino acid variations

**Figure 2:**
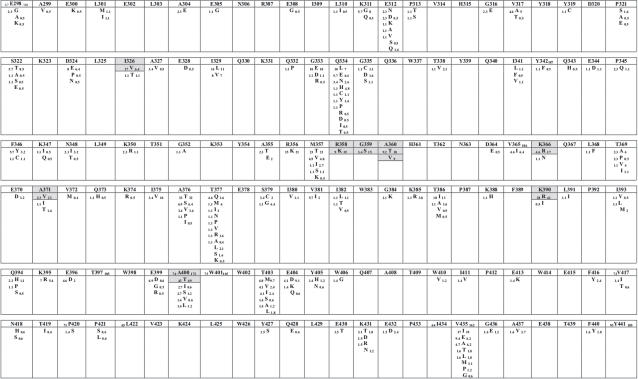
Comprehensive analysis of amino acid sequence variation in HIV-1 RT thumb and connection C-terminal domains showing polymorphic variations between subtype B viruses from drug-naïve and NRTI-treated subjects. Residues are displayed as the subtype B consensus; other residues found among sequences analyzed are shown below each consensus residue. Numbers shown in subscript for each residue represent total number of sequences analyzed (in the consensus sequence) and frequencies in viruses (other residues) from drug-naïve (left) and NRTI-treated (right) subjects. Residues which differed significantly (*p*<0.05) in frequency between naïve and treated patients, as well as the corresponding changed residues, are boxed in gray.

**Figure 3:**
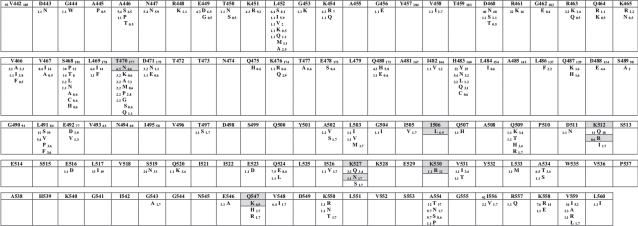
Comprehensive analysis of amino acid sequence variation in HIV-1 RT RNase H C-terminal domains showing polymorphic variations between subtype B viruses from drug-naïve and NRTI-treated subjects. Residues are displayed as the subtype B consensus; other residues found among sequences analyzed are shown below each consensus residue. Numbers shown in subscript for each residue represent total number of sequences analyzed (in the consensus sequence) and frequencies in viruses (other residues) from drug-naïve (left) and NRTI-treated (right) subjects. Residues which differed significantly (*p*<0.05) in frequency between naïve and treated patients, as well as the corresponding changed residues, are boxed in gray.

We analyzed amino acid variations found *in vivo* when comparing NRTI-naïve and treated subjects at each one of 263 codons included in the study. Since a number of amino acid residues are polymorphic across all HIV-1 group M subtypes, we decided to search for NRTI-related mutations only among subtype B sequences available. Subtype B represented alone 51% (260/510) of thumb and connection domain strains and 51% (288/568) of RNase H domain strains in our analysis. The results can be seen in Figures 2 and 3. Fifteen amino acid substitutions had significantly different frequencies when comparing drug-naïve and treatment-experienced subtype B isolates. Of those, 12 had increased prevalence in treated sequences (R358K, G359S, A360T, A360V, K366R, A371V, K390R, A400T, I506L, K527N, K530R and Q547K). The mutations A360V, I506L and Q547K were only seen in subtype B patients under treatment. The largest difference found was for mutation K527N, with a frequency 16 times higher in treated patients (Figure 3). Mutation A371V was seen in 22.6% of treated versus only 2.3% of naïve subtype B isolates (10 times lower). Similarly, mutation K530R was found in 12.1% of treated isolates versus 1.1% of naïve isolates. Another mutation, G359S, was found in 15% of treated isolates, while it was seen only in 3.4% of the drug-naïve isolates (Figure 2). Three amino acid substitutions showed decreased proportions in treatment-experienced subtype B isolates when compared to drug-naïve isolates (I326V, T470N, and K512R). All 15 statistically-significant amino acid differences between drug-naïve and experienced sequences, together with their respective *p*-values, can also be seen in Table 1.

**Table 1:** Frequency of putative NRTI- and AZT-related mutations found among viruses from patients with NRTI or with AZT monotherapy exposure

Mutation	Frequency Naïve	Frequency NRTI-treated	*p*-value	Frequency AZT-treated	*p*-value
I326V	17.2% (15/87)	6.4% (12/188)	**0.008**	7.1% (02/28)	0.157
R358K	9.2% (08/87)	18.7% (35/187)	**0.049**	14.3% (04/28)	0.326
G359S	3.4% (03/87)	15% (28/187)	**0.004**	10.7% (03/28)	0.154
A360T	9.2% (08/87)	17.6% (33/187)	**0.028**	14.3% (04/28)	0.326
A360V	0% (00/87)	9.1% (17/187)	**0.002**	14.3% (04/28)	**0.003**
K366R	4.6% (04/87)	16.7% (31/186)	**0.006**	10.7% (03/28)	0.225
A371V	2.3% (02/87)	22.6% (42/186)	**<0.001**	17.9% (05/28)	**0.009**
K390R	27.6% (24/87)	40.9% (76/186)	**0.043**	60.7% (17/28)	**0.002**
A400T	43.2% (32/74)	69% (119/172)	**<0.001**	71.4% (20/28)	**0.001**
T470N	6.5% (06/93)	0.6% (01/177)	**0.007**	3.7% (01/27)	0.841
I506L	0% (00/93)	6.9% (04/58)	**0.020**	NA**	NA
K512R	8.6% (08/93)	0% (00/58)	**0.024**	NA	NA
K527N	1.1% (01/93)	17.2% (10/58)	**<0.001**	NA	NA
K530R	1.1% (01/93)	12.1% (07/58)	**0.010**	NA	NA
Q547K	0% (00/93)	6.9% (04/58)	**0.020**	NA	NA

* Significant *p*-values at the 0.05 level are boldfaced

** NA, data not available

The role of specific ARV drugs in selecting the mutations depicted here is not easily determined, since the vast majority of treated patients whose viral samples were analyzed have been subjected to multiple, complex drug combinations prior to sample collection. In our dataset, none of the connection domain sequences analyzed was from patients in use of NNRTI, but a small fraction of the RNase H viral sequence (8.6%; 16/185) had previous exposure to that drug class. Therefore, we cannot completely rule out the possibility of a potential influence of NNRTI in the selection of mutations characterized here. However, a limited number of those samples (n = 28) are from patients older in the epidemic and exclusively subjected to AZT monotherapy. We have compared these sequences with those from the drug-naïve dataset in the positions pointed out above. It noteworthy that comparisons were possible only up to RT codon 500, as scarce sequence information is available after that codon for those viral sequences. The comparison can be seen in Table 1. Among the 10 mutations analyzed, 4 were significantly found more frequently in AZT-exposed subjects (A360V, A371V, K390R and A400T). These mutations are expected to be selected by AZT, and perhaps by other NRTI, particularly thymidine analogues.

**Figure 4:**
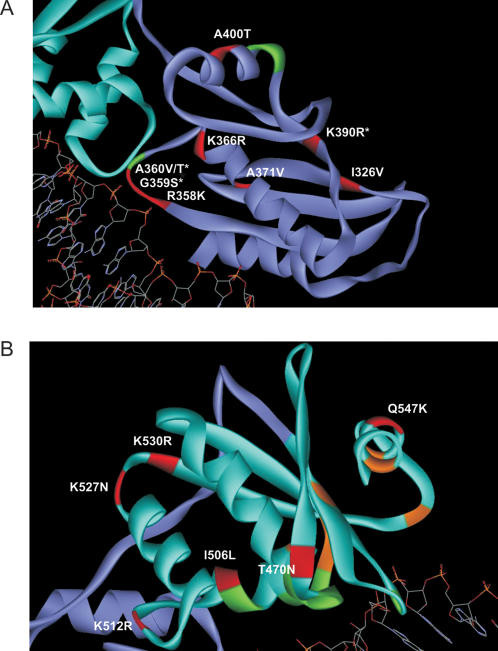
Three-dimensional structure of HIV-1 RT connection (*A*) and RNase H (*B*) domains in contact with template viral DNA showing codons significantly associated with NRTI treatment. The atomic coordinates of HIV-1 RT isolate BH10 (entry 1HYS of the Brookhanven Protein Data Bank) was downloaded into the visualization software ViewerLite v. 5.0 and then manually colored. Subtype B-related substitutions with statistical significance are shown in red. Amino acid residues with asterisks denote those which interact with nucleic acid. Additional residues which interact with nucleic acid, but not found to be selected by treatment in our analysis, are shown in green. Amino acid residues depicted in orange in panel (*B*) represent the RNase H catalytic site.

Mutations subjected to selective pressure were highlighted onto the structural C-terminal domains of HIV-1 RT (Figure 4). In the connection domain, nine mutations were selected by treatment in subtype B. The RNase H domain harbored 6 mutations selected by treatment in this subtype. Interestingly, some of these residues lie in motifs known to contact the nucleic acid (G359S, A360T/V and K390R). As expected, the seven amino acid residues that comprise the RNase H catalytic site (D443, E478, D498, S499, H539, N545 and D549) were highly conserved and remained unchanged under NRTI treatment.

## Discussion

The present study determined the conserved amino acid positions of thumb, connection and RNase H domains of HIV-1 group M RT. The comparison of polymorphisms between drug-naïve and NRTI-experienced patients has evidenced a total of 86 (33%) invariable sites common to both groups. Other studies recently published on the polymorphism of PR (codons 1-99) and RT N-terminal domains (1-320) observed highly conserved sites in approximately half of PR [21] and in over half of RT N-terminal domain [22], independent of treatment. In both studies, the inclusion of non-B subtypes was negligible, with contributions of only 3.9% in the former study and 4.8% in the latter. The use of few non-B subtypes in those analyses may explain the high conservation of PR and RT enzymes as the standard consensus for resistance studies is the subtype B. In our study, we found that one third of RT C-terminal sites are highly conserved, independent of HIV-1 group M subtypes or treatment status.

Regions of C-terminal RT domains lying closer to the DNA:RNA hybrid were found conserved, including amino acid residues that interact with the primer DNA (G359, A360, H361, K395, E396, T473, N474, Q475, K476, Y501 and I505) and the template RNA strand (K390, R448, N474, Q475, Q500 and H539) [20]. Site-directed mutagenesis of RNase H positions N474, Q475 and Y501 rendered the mutant enzymes inefficient in the removal of the PPT primer and modified the RNase H cleavage specificity [23]. In addition, changes at RNase H positions Q475 and Y501 produced defective viruses [24], [25]. The essential role of those positions for RNase H is also reflected in our study, where they were found highly conserved. Positions T473, Q500 and I505 also had a high degree of conservation. Other two positions (R448 and K476) showed a low variability (2–5% variation).

The motif ETFYVDGA spanning RT codons 438-445 was highly conserved (0–5% variation), which is consistent with the fact that it overlaps the protease cleavage site AETFYVD (codons 437-443) located between the connection and the RNase H domains. Mutagenesis experiments in this motif caused defects in virion RT and integrase processing [26]. Positions E438, T439, Y441 and V442 were highly invariable (variation ≤1%) (Figure 1). Positions F440 and D443 presented a low degree of variability (variation 2–5%). Position A437 was the most variable (14%). The impact of this polymorphism on the efficiency of cleavage remains to be experimentally determined.

Another conserved region comprises codons 492-511 and represents the RNase H β4 and αB secondary structures [27]. Amino acids D498, which interacts with divalent cations, and S499, which is also part of the RNase H catalytic site, lie in this motif. The motif 535-553 is also conserved, which harbors the invariable amino acids N545, H539 and D549, this last aspartic acid also interacting with divalent cations. The five residues D498, S499, H539, N545 and D549 comprise the RNase H catalytic site together with two other residues, D443 and E478 [27]. All positions were highly conserved with the exception of position D443, with 2% variation found naturally in drug-naïve patients, but highly conserved in treated isolates.

The function of the connection domain has not been fully characterized. The crystal structure of HIV-1 RT has shown five positions of the connection domain that interact with DNA (G359, A360, H361, K395 and E396) and a unique position (K390) that interacts with RNA [20]. These positions are part of the RNase H primer grip motif, which helps positioning the template:primer at the RNase H active site. More recently, site-directed mutagenesis experiments in all those codons showed individual effects on reducing the RNase H activity either by altering the consensus 2-LTR circle junctions (H361 and E396) or by inserting tRNA primers at those junctions during reverse transcription (A360, H361, T362, K395 and E396) [12]. All mutations decreased the viral replicative capacity including mutations in residues G359 and K390. The authors suggested that these mutations weaken the binding of RT to nucleic acid, therefore affecting RNase H activity. In contrast to the high conservation seen in residues that contact nucleic acids in the RNase H domain, only two residues were highly conserved in the connection domain (H361 and T362; Figure 1). This can be explained by the proximity of these residues to the RNase H catalytic site [12]. The remaining residues presented natural variations within HIV-1 group M (10–70% variation). It is interesting to note that position 390 in the group M consensus is an arginine, where in subtype B it is a lysine. Moreover, the mutation K390R appears to be selected by NRTI usage in subtype B (Figure 4). The role of specific amino acid residues at this position and their impact on the primer grip function deserves further investigation.

The most conserved region of the connection domain encodes amino acids 387-430, where 38 codons (86%) presented high conservation (0–4% variation). This is an interesting finding because this region contains one of two RT sites which interact with the viral integrase (codons 387-422) [28]. Within this motif, 64% (23/36) of the codons had a high degree of conservation among HIV-1 group M strains. All conserved regions presented here are potential targets for new drugs aiming to inhibit HIV-1 group M replication.

When we compared subtype B naïve and treatment-experienced sequence groups, differences between them were found. We discovered 15 mutations related to NRTI treatment. Until now, all studies published on HIV RTI drug resistance relied on the N-terminal half of RT, the target of both RTI drug classes. Such studies neglected the role of C-terminal RT domains in drug resistance. In 2005, Nikolenko *et al.* evidenced the importance of the RNase H domain to drug resistance. They showed that mutations which decreased *in vitro* RNase H activity greatly increased resistance to AZT and d4T [11]. In the present study, we did not find any of the RNase H mutations pointed out in the literature that decreased RNase H activity *in vivo*. However, three of the mutations described here (A360V, I506L and Q547K) were present only in NRTI-experienced isolates. Mutation A360V lies in a residue that contacts nucleic acid, while mutation I506L is close to two residues of the RNase H primer grip [20]. We think that these changes may weaken the RT-DNA:RNA contact, therefore decreasing RNase H activity, while still maintaining virus viability *in vivo*. Such hypothesis was corroborated by recent work of Delviks-Frankenberry and colleagues [29], through alanine-scanning mutagenesis in RNase H primer grip residues. The authors showed concomitant decrease of RNase H activity and increase of AZT resistance by knocking out residues G359, A360, and K390. Each of these mutations did not confer resistance by itself, but in combination with thimydine analog mutations the fold change in resistance to AZT was from 2.5 to 6.5 times higher when compared to viruses carrying only TAMs [29]. All these residues are described herein as related to NRTI treatment.

In view of potential biases created by differences in subtype distribution in drug-naïve and NRTI-experienced sequence groups, we conducted an exclusive analysis of subtype B, the one with the largest number of isolates sequenced, particularly those on NRTI treatment. A small number of NNRTI-experienced patients was present in the dataset, and as possible interactions between NRTI- and NNRTI-selected mutations is known to occur, we cannot completely discard a putative effect of NNRTI in the selection of mutations described here. Nine changes in the connection domain and 6 in the RNase H domain were found related to treatment (Figures 2 and 3). Some changes are within (G359S, A360V/T and K390R) or close (R358K and A400T) to the primer grip, while another is close to the RNase H catalytic site (Q547K; Figure 4). Experimental evidence for the role of those residues in drug resistance is just starting to emerge, but is rapidly accumulation. Nikolenko and colleagues have recently identified eight mutations in the connection domain (E312Q, G335C/D, N348I, A360I/V, V365I, A376S) that increase the resistance to AZT in phenotyping assay [30]. In our analysis, mutation A360V was found significantly associated with NRTI treatment. Interestingly, the study by Nikolenko *et al*. also reported that the reversion of mutation I326V increased AZT resistance [30]. Here, the decrease in frequency of this mutation was associated with treatment (Figures 2 and 3). Additionally, Cane *et al*. correlated some mutations in the RT connection domain with TAMs, G359S, A360V/T and A371V [31]. Once more, all those changes were evidenced in our analyses.

Among the NRTI-related mutations characterized herein, four of them have been associated with AZT in patients with exclusive exposure to that drug. Those included mutations A360V and A371T, found in patients with TAMs [32]. We may speculate that, in addition to AZT, other thymidine analogues (such as d4T) may also select for those mutations, but additional experimental and analytical data is necessary to confirm this hypothesis.

This work was the first to comprehensively evaluate genotypic variation in the C-terminal domains of RT in the context of drug resistance *in vivo* and in most HIV-1 subtypes. The findings corroborate data by other studies [11], [29]–[30] and expand our knowledge about the influence of C-terminal RT domains in antiretroviral drug resistance. We also elaborated a panel of conserved residues for HIV-1 group M independent of treatment which we believe is useful for the design of new drugs that target those domains. The role of RT mutations characterized herein is still undetermined, but they may become pivotal in developing optimized genotyping HIV drug resistance algorithms.
